# CD4+CD25+CD127-Foxp3+ and CD8+CD28- Tregs in Renal Transplant Recipients: Phenotypic Patterns, Association With Immunosuppressive Drugs, and Interaction With Effector CD8+ T Cells and CD19+IL-10+ Bregs

**DOI:** 10.3389/fimmu.2021.716559

**Published:** 2021-07-15

**Authors:** Mostafa G. Aly, Eman H. Ibrahim, Hristos Karakizlis, Rolf Weimer, Gerhard Opelz, Christian Morath, Martin Zeier, Naruemol Ekpoom, Volker Daniel

**Affiliations:** ^1^ Transplantation Immunology, Institute of Immunology, University Hospital Heidelberg, Heidelberg, Germany; ^2^ Nephrology Unit, Internal Medicine Department, Assiut University, Assiut, Egypt; ^3^ Clinical Pathology Department, South Egypt Cancer Institute, Assiut University, Assiut, Egypt; ^4^ Department of Internal Medicine, University of Giessen, Giessen, Germany; ^5^ Department of Nephrology, University Hospital Heidelberg, Heidelberg, Germany

**Keywords:** Tregs, Bregs, renal transplantation, CD8+ Tregs, IL-10+ Bregs, CD8+CD28- Tregs

## Abstract

**Introduction:**

Gaps still exist regarding knowledge on regulatory cells in transplant recipients. We studied the phenotypic patterns of CD4+, CD8+CD28- Tregs, and CD19+IL-10+ Bregs in the blood of healthy controls (HC), end-stage kidney disease patients (ESKD), early and late stable renal transplant recipients (Tx), and transplant recipients with steroid-treated acute cellular rejection 1 week–3 months after successful treatment. We also investigated the relationship between immunosuppressive drugs and the aforementioned regulatory cells in transplant recipients.

**Methods:**

We recruited 32 HC, 83 ESKD, 51 early Tx, 95 late Tx, and 9 transplant patients with a recent steroid-treated acute cellular rejection. Besides CD19+IL-10+ Bregs, we analyzed absolute and relative frequencies of CD4+CD25+CD127-Foxp3+ Tregs and CD8+CD28- Tregs and their expression of IL-10, TGF-ß, IFN-g, and Helios.

**Results:**

We found a negative correlation between absolute CD4+CD25+CD127-Foxp3+ Treg and relative CD19+IL-10+ Breg frequencies in early Tx recipients (r=-0.433, *p*=0.015, n=31). In that group, absolute CD4+CD25+CD127-Foxp3+ Tregs were negatively associated with steroid dose and tacrolimus trough levels (r=-0.377, *p* = 0.021, n=37; r=-0.43, *p*=0.033, n=25, respectively), opposite to IL-10+ Bregs, whose frequency apparently was not negatively affected by potent immunosuppression early posttransplant. We found also lower CD4+CD25+CD127-Foxp3+ Tregs in patients treated with basiliximab or rATG as compared with ESKD patients (*p*=0.001 and *p <*0.001, respectively). No difference in absolute IL-10+ Bregs could be detected among these 3 patient groups. Early Tx recipients showed lower CD4+CD25+CD127-Foxp3+ Tregs within 3 months of antibody induction than after 3 months (*p* = 0.034), whereas IL-10+ Bregs showed higher relative counts during the first 3 months post antibody induction than after 3 months (*p* = 0.022). Our findings suggest that IL-10+ Bregs decrease with time posttransplantation independent of the effect of antibody induction and dose of other immunosuppressive drugs.

**Conclusion:**

These findings suggest that CD19+IL-10+ Bregs and CD4+CD25+CD127-Foxp3+ Tregs behave in opposite ways during the early posttransplant period, possibly due to a predominant negative impact of high doses of immunosuppressants on Tregs. CD19+IL-10+Bregs do not seem to be suppressed by antibody induction and early potent immunosuppression with chemical drugs.

## Introduction

Renal transplantation represents the modality of choice for end-stage kidney disease (ESKD) patients. Since chemical immunosuppressants expose transplant recipients to an increased risk of serious infections and development of malignancy, specific cell therapy using regulatory cells with well-established stable phenotypes and proven immunosuppressive potential might help to minimize the use of immunosuppressants in solid organ transplant recipients. Tregs have been the focus of extensive research. CD4+ Tregs display the marker combination CD4+CD25+CD127-Foxp3+ and play a key role in ameliorating autoimmune diseases, preventing allograft rejection, and maintaining peripheral tolerance. CD4+ Tregs originate in the thymus (tTregs) or can be generated in the peripheral blood (pTregs) or *in vitro* (iTregs) upon induction of naïve CD4+CD25- T cells with IL-2 and TGF-ß (1). Unlike tTregs, which express Foxp3 permanently, pTregs and iTregs express Foxp3 transiently and can reprogram to effector cells. So far, no consensus has been reached regarding a specific marker that distinguishes tTregs from pTregs. Although Helios, a member of the Ikaros transcription factors family, was proposed as a marker of tTregs but not pTregs, this suggestion has been contradicted (2, 3). Helios-expressing Tregs were shown to exhibit stable Foxp3 and possess potent suppressive potential *in vitro* (4). tTregs were shown to possess completely demethylated Treg-specific demethylated region (TSDR), contrary to pTregs, which exhibit partially demethylated TSDR (5, 6). Among other mechanisms, CD4+ Tregs exert their function through production of inhibitory cytokines, including IL-10 and TGF-ß1. Similar to CD4+ Tregs, CD8+ T cell subsets with immunosuppressive potential were identified. CD8+CD28- Tregs represent the predominant type of CD8+ regulatory cells in humans. Among other mechanisms, CD8+CD28- Tregs were found to exert their function by induction of tolerance in antigen presenting cells (APC) with subsequent inhibition of CD4+ T cells (7).

B cells have long been known for antibody production and antigen presentation. A study showed that induction with rituximab, a CD20-depleting agent, in renal transplant recipients led to an increased incidence of acute rejection. This was attributed to the depletion of regulatory B cells (Bregs) along with effector B cells (8). Unlike CD4+ Tregs, to date, no consensus on a marker combination to distinguish different Bregs has been reached. IL-10+ B cells represent the most extensively studied Bregs. IL-10+ Bregs exert their function through stimulating Tregs and promoting the conversion of CD4+CD25- effector T cells to Tregs (9). Blocking of IL-10 or IL-10 receptors in IL-10+ Bregs did not completely abrogate their suppressive potential (10).

Understanding the interaction of CD4+CD25+CD127-Foxp3+ Tregs, CD8+CD28- Tregs, and CD19+IL-10+ Bregs with one another and their relationship with immunosuppressants in renal transplant recipients is indispensable to identify subsets of regulatory cells with stable phenotypes and limited reprogramming potential that can be used for cell therapy. In the current study, we aimed to further characterize CD4+ and CD8+CD28- Tregs according to expression of surface markers and transcription factors, including IL-10, TGF-ß1, IFN-g, and Helios in HC, ESKD patients on regular dialysis, early and late stable renal transplant recipients, and transplant recipients with a relatively recent steroid-treated acute cellular rejection (STCR). In addition, we analyzed the association between standard immunosuppressants and CD4+CD25+CD127-Foxp3+ Tregs, CD8+CD28- Tregs, and IL-10+ Bregs. Moreover, we studied the association between CD4+CD25+CD127-Foxp3+ Tregs, CD8+CD28- Tregs, and IL-10+ Bregs in the aforementioned study groups. Lastly, we studied the association between CD4+CD25+CD127-Foxp3+, CD8+CD28- Tregs, or IL-10+ Bregs and graft function in renal transplant recipients. To the best of our knowledge, our study is the first to analyze the association of CD4+CD25+CD127-Foxp3+ Tregs, CD8+CD28- Tregs, and IL-10+ Bregs in early and late renal transplant recipients in relation to standard initial and maintenance immunosuppression.

## Materials and Methods

### Healthy Volunteers and Patients

In the current cross-sectional study, thirty-two healthy volunteers from blood donors and staff members served as controls (HC). Blood samples from eighty-three ESKD patients on regular dialysis, fifty-one early renal Tx recipients (within one year post transplantation), ninety-five late renal Tx recipients (more than a year post transplantation), and nine renal Tx recipients with a relatively recent biopsy-proven steroid-treated acute rejection (STCR) (one week to 3 months before blood sampling) were obtained once from each patient in the period between June 2015 and November 2019 from the Department of Nephrology of Heidelberg University and the Internal Medicine Department of Giessen University. ESKD patients were included in the study upon fulfilling the following criteria: age > 18 years, no previous transplants, and no current treatment with immunosuppressive drugs. Renal Tx patients were included if they fulfilled the following criteria: age > 18 years and solitary ABO- and HLA-compatible kidney transplantation. Exclusion criteria included age below 18 years, combined organ Tx, sensitized patients who received desensitization prior to and directly after Tx, previous treatment with rituximab, current treatment with belatacept, and STCR patients treated with rabbit ATG (rATG) in addition to pulse steroid. HC as well as patients gave written informed consent for the tests conducted in this study. The current study was approved by the Heidelberg ethical committee (S-225/2014) and was conducted in accordance with the Declaration of Helsinki. **Table 1** shows the demographic data of the patients.

**Table 1:** Demographic data of healthy controls (HC), end-stage renal disease patients (ESKD), early renal transplant recipients (Tx), late renal transplant recipients, and steroid-treated renal transplant patients with acute cellular rejection (STCR).

	HC (n=32)	ESKD (n=83)	Early Tx (n=51)	Late Tx (n=95)	STCR (n=9)
**Age (years, median, IQR)**	45 (37-53)	51 (43-62)	50 (43-59)	58 (49-65)	55 (48-68)
**Sex (%)**					
** Female**	53	40	35	32	11
** Male**	47	60	65	68	89
**Days post-transplant (median, IQR)**	–	–	38 (14-109)	2030 (1742-4340)	61 (20-440)
**Type of donor (%)**					
** Living**	–	–	24	39	11
** Deceased**	–	–	76	61	89
**Graft No. (%)**					
** First**	–	–	92	94	89
** Second**	–	–	6	3	11
** Third**	–	–	2	3	–
**Delayed graft function (%)**	–	–	8	10	12.5
**Cold ischemia time (minutes, median, IQR)**	–	–	726 (488-970)	510 (117-908)	644 (515-991)
**GFR ml/min (median, IQR)**	–	–	39 (26-59)	52 (34-69)	25 (13-43)
**Previous CMV infection (%)**	–	–	24	20	22
**Previous BKVN (n, %)**	–	–	5	12	0
**Patients with previous rejection (%)**					
** Yes**	–	–	6	21	100
** No**	–	–	94	79	0
**Protein/creatinine ratio g/mol (median, IQR)**	–	–	24 (13-37)	17 (8-59)	30 (17-115)
**CRP mg/l (median, IQR)**	–	9 (2-32)	10 (2-21)	11 (2-52)	3 (2-10)
**Etiology of the end stage renal disease (%)**					
**Chronic glomerulonephritis**	–	38	41	39	33.5
**Diabetes**	–	10	12	5.5	11
**Hypertension/ischemic**	–	2	2	4.5	11
**ADPKD**	–	17	12	17	11
**Hereditary/congenital**	–	5	8	6.5	11
**Others**	–	15	14	16.5	22.5
**Unknown**	–	13	12	11	0
**Antibody induction (%)**					
**Basiliximab**			78	93	87
**ATG**			22	7	13
**Maintenance immunosuppression (% of patients)**					
**Cyclosporine**	–	–	48	21	56
**Tacrolimus**	–	–	52	73	33
**MMF**	–	–	97	63	100
**Steroids**	–	–	98	68	100
**Everolimus**	–	–	0	4.5	11
**Azathioprine**	–	–	0	9	0
**Cyclosporine trough level (ng/ml)**			167 (142-201)	79 (70-108)	202 (187-223)
**Tacrolimus trough levels (ng/ml)**			9 (7 -10.7)	6 (5- 7.6)	5.5 (4.6-7.6)

HC, healthy control; ESKD, end-stage kidney disease; Tx, transplant recipients; IQR: Interquartile range; CMV, cytomegalovirus; BKVN polyoma virus nephropathy, GFR, glomerular filtration rate; ADPKD, Autosomal dominant polycystic kidney disease; MMF, mycophenolate mofetil.

### Determination of Absolute Count of Lymphocyte Subsets

To calculate the absolute count of lymphocyte subsets, 50 μl of whole blood was incubated with fluorochrome-labeled monoclonal antibodies against CD3, CD4, CD8, CD45 (Cat. no. 342417), CD19, and CD16+CD56 (Cat. no 342416) (all from BD Biosciences). After vortexing and incubation in the dark at room temperature for 15 minutes, 500 µl BD FACS Lyse solution (1:10) was added. The tubes were then vortexed and incubated in the dark at room temperature for 15 minutes. Finally, all samples were evaluated with four-color FACSCalibur II double-laser flow cytometer (BD Biosciences). At least 100,000 events were analyzed in the initial FSC/SSC dot plot.

### Identification of CD4+ CD25+CD127-Foxp3+ Tregs, CD8+CD28- Tregs, IL-10+ Bregs, Perforin+, and Granzyme B+ CD8+ T Cells

**Figure 1:**
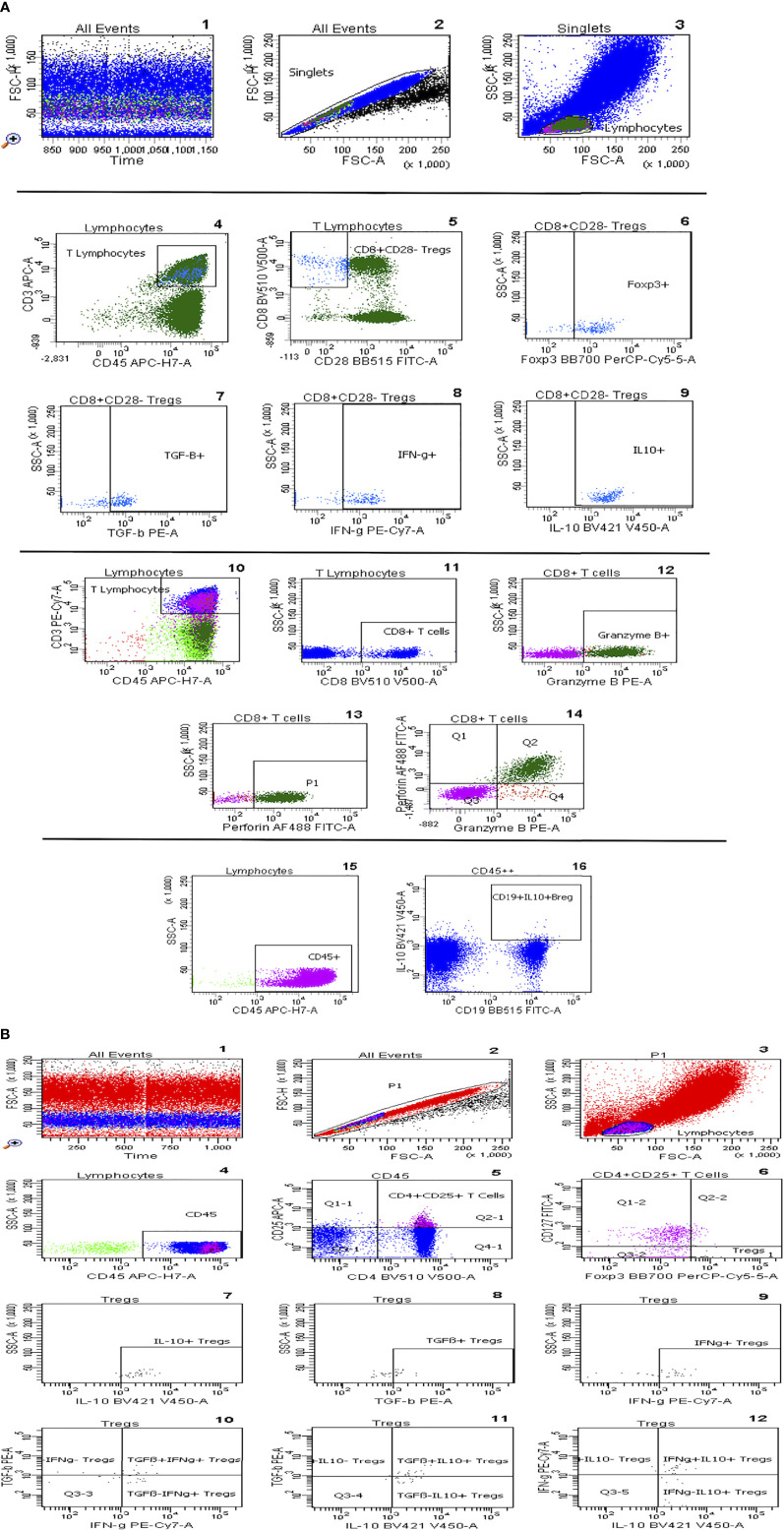
**(A)** Gating strategy of CD8+CD28- Tregs, IL-10+ Bregs, and perforin+ and/or granzyme B+ CD8+ T cells. After ensuring the stability of the run, selection of singlets and gating of lymphocytes according to their size (1-3), T-lymphocytes were gated based on CD45 *vs* CD3 plot, CD8+CD28- Tregs were then selected and their subsets, Foxp3+, TGF-β+, IFN-g+ and IL-10+, were gated respectively (4-9). CD8+ T cells are gated out of T cells and then Granzyme B+ and Perforin+ CD8+ T cells were selected (10-14). IL-10+CD19+ regulatory B cells are gated out of CD45+ cells (15,16). **(B)** Gating strategy of CD4+CD25+CD127-Foxp3+ Tregs: Graph 1 includes all events analysed in the tube. Graph 2 exhibits the exclusion of doublets. Graph 3 shows FSC *vs* SSC dot plot and gating of lymphocytes according to size, CD45 *vs* SSC dot plot permits elimination of debris and focuses on lymphocytes (Graph 4), CD25 *vs* CD4 dot plot allows the identification of CD4+CD25+ T lymphocytes (Graph 5), the Foxp3+CD127- cluster was gated out of CD4+CD25+ lymphocytes and identifies Tregs (graph 6). In addition, graphs 7-12 show Treg subsets gated out of total Tregs.

Flow cytometric determinations were performed immediately after arrival of the blood samples in the lab. Fluorochrome-labeled monoclonal antibody against CD45 (5 µl; BD cat. no. 560178), CD19 (5 µl; BD cat. no. 564456), CD25 (20 µl; BD cat. no. 555434), CD127 (20 µl; BD cat. no. 560549), CD4 (5 µl; BD cat. no. 562970), CD3 (5 µl; BD cat. no. 563423), CD8 (5 µl; BD Cat. no 564492), and CD28 (5 µl; BD Cat. no 564492) were added to the tubes, whereas Foxp3 (5 µl, BD cat. no. 566526), IL-10 (5 µl; BD Cat. no. 564053), Helios (5 µl; BD Cat. no. 563801), IFN-g (5 µl; BD Cat. no. 557643), TGF-β1 (5 µl; BD Cat. no. 562339), perforin (5 µl; BD Cat. no. 563764), and granzyme B (5 µl; BD Cat. no. 561142) were not added until the permeabilization process had been performed. 200 µl of whole heparinized blood was added to each tube. All tubes were vortexed briefly and incubated at room temperature in the dark for 30 minutes. Afterwards, 2 ml of a 1:10 diluted Lyse solution from BD Biosciences was added to all tubes. The tubes were vortexed, incubated at room temperature in the dark for 10 minutes, and then centrifuged at 1300 rpm for 8 minutes. Thereafter, supernatant was discarded, 1.5 ml PBS was added, and tubes were vortexed again briefly. Then, tubes were centrifuged at 1300 rpm for 8 minutes and supernatant was discarded. Five hundred µl of 1:10 diluted BD Permeabilizing II solution was added to the tubes. After 10 minutes incubation, 1.5 ml PBS was added. Tubes were vortexed briefly and subsequently centrifuged at 1300 rpm for 8 minutes. The supernatant was removed and discarded. Antibodies against the intracellular determinant, Foxp3, were added to the pellets. After 30 minutes incubation in the dark at room temperature, 1.5 ml PBS was added. Tubes were vortexed again briefly and subsequently centrifuged at 1300 rpm for 8 minutes. The supernatant was removed and discarded. Finally, 100 µl PBS was added to the pellets. Then cells were analyzed. All samples were evaluated with eight-color fluorescence using the FACSCanto II triple-laser flow cytometer (BD Biosciences). At least 100,000 events were analyzed in the initial FSC/SSC dot plot. The gating strategy is depicted in **Figure 1**. The relative counts of CD4+CD25+CD127+ Tregs, CD8+CD28- Tregs, and IL-10+ Bregs were estimated in relation to the total CD45+ lymphocyte pool unless otherwise mentioned.

### Statistical Analysis

In the current study, normally distributed data were presented as mean ± standard deviation (SD), whereas skewed data were presented as median and interquartile range. Mann-Whitney or Kruskal-Wallis test with Dunn’s multiple comparisons test were conducted to test for differences between scale parameters among different study groups. Pearson’s correlation coefficient or Spearman rank correlation test was applied, according to the distribution of data, to show the association between various parameters. Multivariate regression analysis with backward elimination method was conducted to test whether CD4+CD25+CD127-Foxp3+ Tregs, CD8+CD28- Tregs, or CD19+IL-10+ Bregs were independently associated with the doses of standard immunosuppressive drugs in early transplant recipients. Statistical analysis was performed using IBM SPSS statistics 26 (IBM, Ehningen, Germany). *P*-values < 0.05 were considered significant.

## Results

### CD4+ T, CD8+ T, and CD19+ B Cells in Healthy Controls (HC), End-Stage Kidney Disease (ESKD), Early and Late Transplant Recipients (Tx), and Transplant Recipients With a Relatively Recent Steroid-Treated Acute Cellular Rejection (STCR)

As shown in **Table 2**, compared with HC, ESKD patients, early and late Tx recipients, and STCR patients showed significantly lower absolute CD4+ T cell counts (*p* = 0.012; *p*= 0.001; *p*= 0.01; *p*= 0.002, respectively). Early and late Tx recipients did not show a significant difference regarding the absolute or relative CD4+ T cell counts (*p* = 1; *p*= 0.956, respectively). Likewise, the absolute CD8+ T cell counts were significantly lower in ESKD patients and early Tx recipients compared with HC (*p* = 0.005; *p*= 0.001, respectively), and did not differ significantly from those in late Tx and STCR patients (*p* =NS). The late Tx recipients showed significantly higher relative CD8+ T counts compared with HC, ESKD patients, and early Tx recipients (*p* = 0.042; *p*<0.001; *p*= 0.019, respectively). Opposite to CD8+ T cells, CD19+ B cell relative counts were significantly lower in late Tx recipients compared to early Tx recipients. In summary, CD4+ T cells did not differ significantly between early and late Tx recipients. CD8+ T cells were higher in late than in early Tx recipients, whereas the relative CD19+ B cell counts were higher in early than in late Tx recipients.

**Table 2:** Comparison of the absolute and relative counts of CD4+, CD8+ T cells, CD4+CD25+CD127-Foxp3+ and CD8+CD28- Tregs, IL-10+CD19+ B, perforin+, and granzyme B+ CD8+ T cell subsets in healthy controls (HC), end-stage kidney disease patients (ESKD), early and late renal transplant (Tx) patients, and renal transplant patients with a relatively recent steroid-treated acute cellular rejection (STCR).

	HC (n=32)	ESKD (n=83)	Early Tx (n=51)	Late Tx (n=95)	STCR (n=9)	P
**CD3+/µl**	1254 (1010-1585)	861 (589-1129)	766 (362-1165)	1010 (707-1425)	630 (270-986)	<0.001
**%CD3+**	70 (66-76)	75 (68-83)	73 (63-81)	81 (72-87)	72 (60-83)	<0.001
**CD4+/µl**	866 (657-940)	621 (348-796)	511 (220-777)	592 (377-815)	230 (139-649)	<0.001
**%CD4+**	42 (41-49)	49 (43-56)	50 (39-57)	46 (36-51)	35 (33-43)	0.002
**CD8+/µl**	369 (305-552)	254 (159-360)	223 (128-370)	418 (233-544)	211 (98-394)	<0.001
**%CD8+**	22.5 (18-29)	20 (16-28)	22 (18-31)	30 (20-38)	31 (17-44)	<0.001
**CD19+/µl**	180 (116-221)	35 (17-94)	84 (47-160)	46 (16-88)	33 (18-90)	<0.001
**%CD19+**	9.8 (6-11)	3 (2-9)	9 (6-14)	4 (1-6)	4 (1.7-12)	<0.001
**CD4+ Treg/μl**	20 (15-27)	13 (10-18)	6 (4-12)	9 (4-18)	2.7 (1.5-10.5)	<0.001
**%CD4+ Treg**	1.1 (0.8-1.5)	1.2 (0.8-1.6)	0.7 (0.4-1.2)	0.6 (0.4-1)	0.5 (0.3-0.6)	<0.001
**CD8+CD28- Tregs/µl**	84 (63-239) (n=16)	68 (36-186) (n=22)	38 (14-121) (n=36)	149 (42-323) (n=45)	81 (29-250)	0.012
**%CD8+CD28- Tregs**	6 (3-12) (n=16)	9 (3.6-19) (n=22)	5 (2-11) (n=36)	13 (5-24) (n=45)	17.6 (6-25)	0.038
**CD19+IL-10+ Bregs/µl**	9 (6-13) (n=29)	4 (2-13) (n=19)	9 (5-12) (n=31)	4.5 (2-10) (n=30)	4.7 (2-9)	0.005
**% CD19+IL-10+ Bregs**	0.5 (0.3-0.6) (n=29)	0.4 (0.2-1) (n=19)	1 (0.6-1.5) (n=31)	0.4 (0.2-0.8) (n=30)	0.6 (0.3-1)	0.001
**Perforin+CD8+/µl**	32 (6 -80) (n=16)	14 (2-38) (n=22)	4 (1-12) (n=36)	18 (7-71) (n=45)	20 (1-49)	0.001
**% Perforin+ CD8+ (within total CD8+ T cells)**	2 (0.3 - 4) (n=16)	1.3 (0.5 - 4) (n=22)	0.5 (0.2 -1.2) (n=36)	2.4 (0.5 - 6) (n=45)	2.3 (0.2 - 7)	0.017
**Granzyme+ CD8+/µl**	60 (29 -123) (n=16)	62 (29 -120) (n=22)	30 (12 - 90) (n=36)	178 (52 - 306) (n=45)	85 (28 - 236)	< 0.001
**% Granzyme+ CD8+ (within total CD8+ T cells)**	3.6 (1 - 7) (n=16)	6.5 (3 - 13) (n=22)	4.5 (1 – 12) (n=36)	13.6 (6 -23) (n=45)	16 (6 - 24)	<0.001

All values are expressed as median and Interquartile range.

% represent the proportion of cells to total lymphocytes (CD45+ cells).

### CD4+CD25+CD127-Foxp3+ Tregs in Healthy Controls (HC), End-Stage Kidney Disease (ESKD), Early and Late Transplant Recipients (Tx), and Transplant Recipients With a Relatively Recent Steroid-Treated Acute Cellular Rejection (STCR)

**Figure 2:**
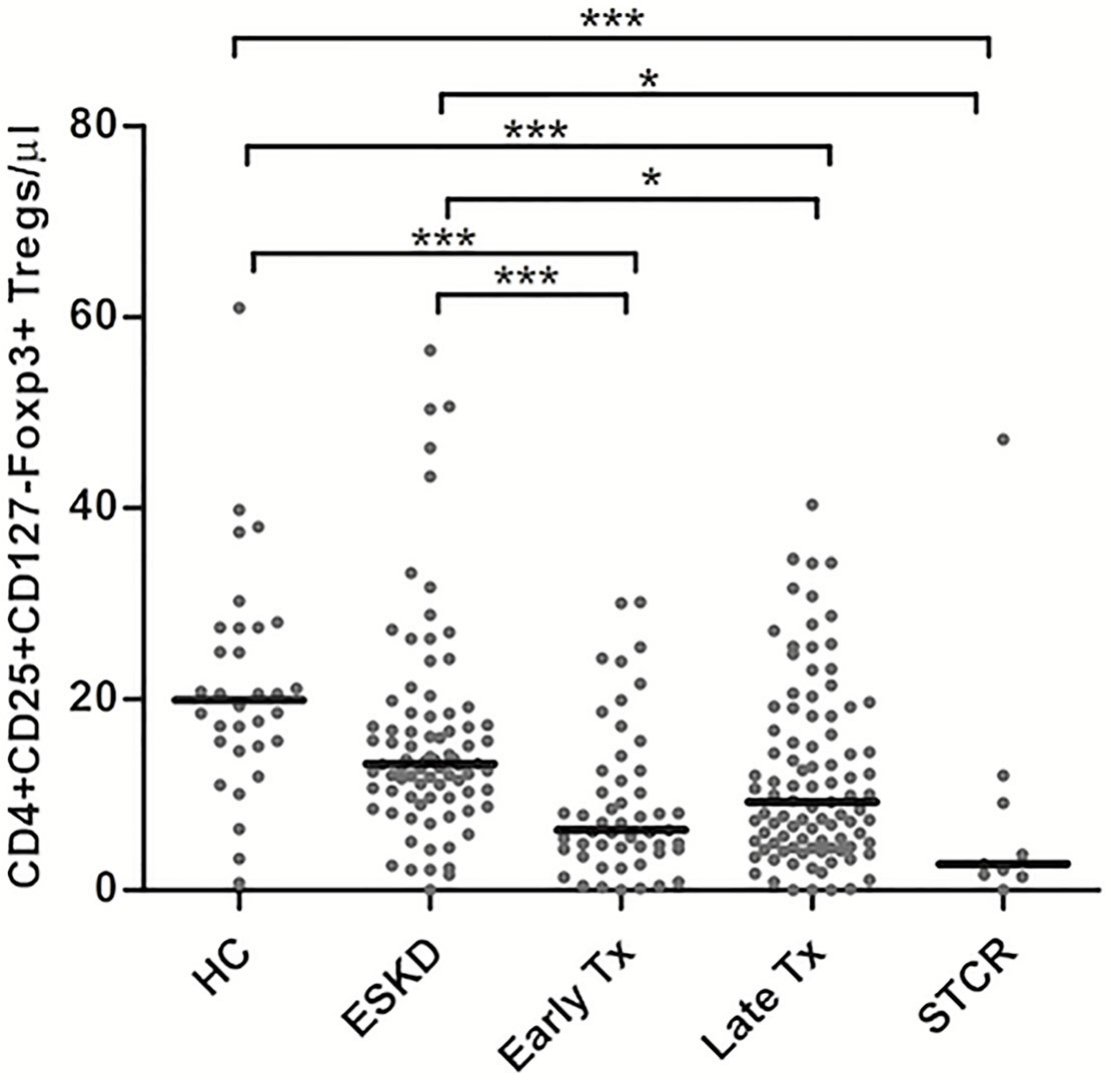
Comparison of the absolute CD4+CD25+CD127-Foxp3+ Treg counts among healthy controls (HC), end-stage kidney disease on regular dialysis (ESKD), early and late transplant recipients (Tx), and transplant recipients with a steroid-treated acute rejection (STCR): Medians were compared among the groups with Kruskal-Wallis test and p-values were adjusted after correction for multiple comparisons, *p < 0.05, ***p < 0.001.

As shown in **Figure 2**, HC showed the highest absolute and relative CD4+CD25+CD127-Foxp3+ Treg counts followed by ESKD patients. In contrast, early Tx and STCR patients showed the lowest absolute and relative Treg counts.

Late transplant recipients showed significantly lower absolute and relative Treg counts compared with HC and ESKD patients (*p <*0.001, *p*= 0.025; *p* = 0.032, *p* = 0.002, respectively). Likewise, early transplant recipients showed significantly lower absolute Treg counts than HC and ESKD patients (*p <*0.001, *p* = 0.002; respectively). We did not find a significant difference in the absolute and relative Treg counts either between HC and ESKD patients or between early and late Tx recipients after correction for multiple comparisons (*p* = 0.2, *p* = 1; *p* = 0.75, *p* = 1; respectively).

### Expression of IFN-g, IL-10, TGF-ß1, and Helios in CD4+CD25+CD127-Foxp3+ Tregs in HC, ESKD, Early and Late Recipients Tx, and STCR Patients

To further characterize the phenotypic pattern of CD4+CD25+CD127-Foxp3+ Tregs, we analyzed their intracellular expression of IFN-g, IL-10, TGF-ß1, and Helios in the study groups. As shown in **Table 3**, we found that the majority of CD4+ Tregs expressed IL-10 in all study groups. Around one third of CD4+ Tregs expressed IFN-g in all study groups, whereas around one half of CD4+ Tregs expressed Helios in ESKD, early, and late Tx patients. Only 10-20% of CD4+ Tregs expressed TGF-ß1 in all study groups.

**Table 3:** Comparison of the absolute and relative counts of IFN-g+, IL-10+, TGF-ß1+, and Helios+ CD4+CD25+CD127-Foxp3+ cell subsets in healthy controls (HC), end-stage kidney disease patients (ESKD), early and late renal transplant (Tx) patients, and renal transplant patients with a recent steroid-treated acute cellular rejection (STCR).

	HC (n=32)	ESKD (n=83)	Early Tx (n=51)	Late Tx (n=95)	STCR (n=9)	*P*-value
**CD4+ Tregs/µl**	20 (15-27)	13 (10-18)	6 (4-12)	9 (4-18)	2.7 (1.5-10.5)	<0.001
**% CD4+ Tregs**	1.1 (0.8-1.5)	1.2 (0.8-1.6)	0.7 (0.4-1.2)	0.6 (0.4-1)	0.5 (0.3-0.6)	<0.001
***IFN-g+ CD4+ Tregs/µl***	7 (4-9) (n=29)	5 (3-8) (n=23)	2 (1-4) (n=37)	3 (1-7) (n=45)	0.6 (0.3-2)	<0.001
**% IFN-g+ CD4+ Tregs**	0.4 (0.3-0.5) (n=29)	0.5 (0.3-0.8) (n=23)	0.2 (01.-0.3) (n=37)	0.3 (0.1-0.5) (n=45)	0.1 (0-0.1)	<0.001
**IL-10+ CD4+ Tregs/µl**	14 (10-21) (n=29)	11 (5-15) (n=23)	4 (1-7) (n=37)	10 (1-17) (n=45)	1.7 (1-7.5)	<0.001
**% IL-10+ CD4+ Tregs**	0.7 (0.6-1.1) (n=29)	1 (0.7-1.5) (n=23)	0.4 (0.2-0.9) (n=37)	0.8 (0.1-1.3) (n=45)	0.2 (0.1-0.4)	0.001
**TGF-ß1+ CD4+ Tregs/µl**	4 (2-6) (n=29)	2 (1-3) (n=23)	0.7 (0.2- 1.6) (n=37)	1 (0.4-2.6) (n=45)	0.4 (0.1-1.4)	<0.001
**%TGF-ß1+ CD4+ Tregs**	0.2 (0.1-0.4) (n=29)	0.2 (0.1-0.4) (n=23)	0.1 (0-0.2) (n=37)	0.1 (0-0.2) (n=45)	0 (0-0.1)	<0.001
**Helios+ CD4+ Tregs/µl**	Not assessed	8 (5-11) (n=60)	4 (2-8) (n=14)	4 (2-5) (n=50)	Not assessed	<0.001
**% Helios+ CD4+ Tregs**	Not assessed	0.6 (0.4-0.9) (n=60)	0.4 (0.2-0.9) (n=14)	0.2 (0.1-0.4) (n=50)	Not assessed	<0.001

All values are expressed as median and Interquartile range.

% represent the proportion of cells to total lymphocytes (CD45+ cells).

### IL-10-Producing CD4+CD25+CD127-Foxp3+ Tregs in HC, ESKD Patients, Early and Late Tx Recipients, and STCR Patients

After correction for multiple comparisons, we found significantly lower absolute and relative CD4+CD25+CD127-Foxp3+IL-10+ Treg counts in early Tx recipients than ESKD patients (*p*= 0.039; *p*=0.003, respectively). Furthermore, the absolute CD4+CD25+CD127-Foxp3+IL-10+ Treg count was significantly lower in early Tx recipients than in HC (*p*=0.001). As expected, STCR patients showed significantly lower absolute CD4+CD25+CD127-Foxp3+IL-10+ Treg counts than HC (*p*=0.008), and they showed significantly lower relative CD4+CD25+CD127-Foxp3+IL-10+ Treg counts than ESKD patients (*p*=0.014) (**Table 3**). These findings suggest a negative impact during the early Tx phase through the administered high doses of immunosuppressive drugs on IL-10-producing CD4+CD25+CD127-Foxp3+ Tregs.

### IFN-g-Producing CD4+CD25+CD127-Foxp3+ Tregs in HC, ESKD Patients, Early and Late Tx Recipients, and STCR Patients

As shown in **Table 3**, both early and late Tx recipients showed significantly lower absolute CD4+CD25+CD127-Foxp3+IFN-g+ Tregs than HC (*p*=0.001; *p*=0.008, respectively). Furthermore, STCR patients showed significantly lower absolute CD4+CD25+CD127-Foxp3+IFN-g+ Tregs than HC and ESKD patients (*p*=0.001; *p*=0.011, respectively). These findings suggest a negative impact early after transplantation, possibly through the administered high doses of immunosuppressants, on IFN-g-producing CD4+CD25+CD127-Foxp3+ Tregs.

### TGF-ß1-Producing CD4+CD25+CD127-Foxp3+ Tregs in HC, ESKD Patients, Early and Late Tx Recipients, and STCR Patients

As shown in **Table 3**, we found significantly lower absolute TGF-ß1-producing CD4+CD25+CD127-Foxp3+ Treg counts in early and late Tx recipients as well as STCR patients compared with HC (*p*<0.001; *p*<0.001; *p*= 0.001, respectively). Likewise, early Tx recipients showed significantly lower TGF-ß1-producing CD4+CD25+CD127-Foxp3+ Tregs compared with ESKD patients (*p*= 0.033).

### Helios-Expressing CD4+CD25+CD127-Foxp3+ Tregs in HC, ESKD Patients, Early and Late Tx Recipients

We found significantly lower absolute and relative Helios+ CD4+CD25+CD127-Foxp3+ Treg counts in late Tx recipients compared with ESKD patients (*p*<0.001; *p*<0.001, respectively).

### CD8+ CD28- Tregs in HC, ESKD, Early and Late Recipients Tx, and STCR Patients

**Figure 3:**
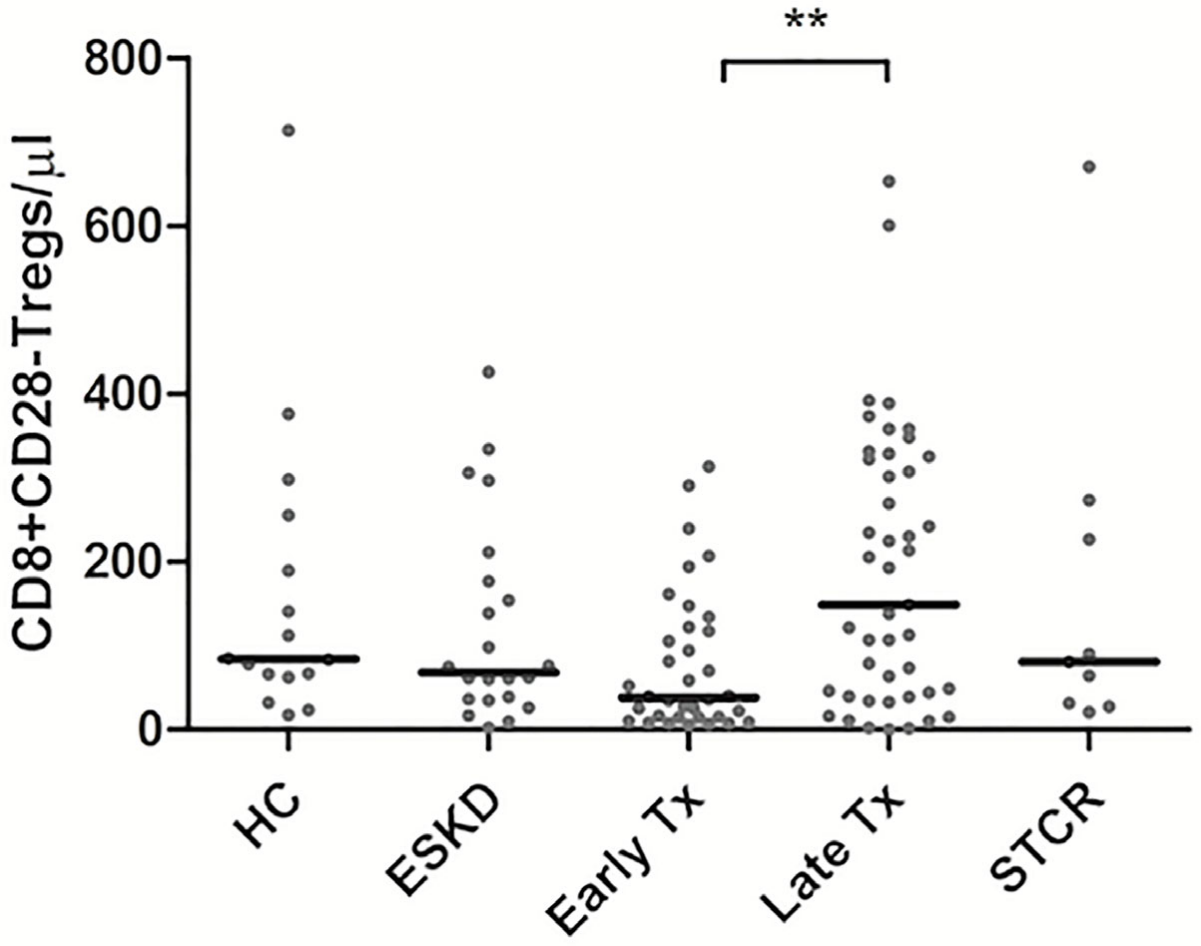
Comparison of the absolute CD8+CD28- Treg counts among healthy controls (HC), end-stage kidney disease on regular dialysis (ESKD), early and late transplant recipients (Tx), and transplant recipients with a steroid-treated acute rejection (STCR): Medians were compared among the groups with Kruskal-Wallis test and p-values were adjusted after correction for multiple comparisons, **p < 0.01.

CD8+CD28- Tregs showed significantly lower absolute counts in early than in late Tx patients (*p* = 0.005) (**Table 4**). As shown in **Figure 3**, we found no statistically significant difference regarding the absolute or relative CD8+CD28- Treg counts (within CD45+ lymphocytes pool) among all other groups except between early and late Tx recipients. Contrary to CD4+Foxp3+ Tregs, early Tx recipients showed significantly lower absolute CD8+CD28- Treg counts than late Tx recipients (*p* = 0.005).

**Table 4:** Comparison of the absolute and relative counts of IFN-g+, IL-10+, TGF-ß1+, and Foxp3+ CD8+CD28- Treg cell subsets in healthy controls (HC), end-stage kidney disease patients (ESKD), early and late renal transplant (Tx) patients, and renal transplant patients with a recent steroid-treated acute cellular rejection (STCR).

	HC (n=16)	ESKD (n=22)	Early Tx (n=36)	Late Tx (n=45)	STCR (n=9)	*P*-value
**Total CD8+CD28- Tregs/µl**	84 (63-239)	68 (36-186)	38 (14-121)	149 (42-323)	81 (29-250)	0.012
**% total CD8+CD28- Tregs**	6 (3-12)	9 (3.6-19)	5 (2-11)	13 (5-24)	17.6 (6-25)	0.038
**IFN-g+ CD8+CD28- Treg/µl**	0.4 (0-0.7)	0.1 (0-0.3)	0 (0-0.1)	0.1 (0-0.2)	0 (0-0)	0.009
**% IFN-g+ CD8+CD28- Treg**	0 (0-0)	0 (0-0)	0 (0-0)	0 (0-0)	0 (0-0)	0.04
**IL-10+ CD8+CD28- Treg/µl**	0.7 (0.1-4)	2.4 (0.1-9)	1.7 (0.1-3)	4.6 (1-10)	0.6 (0.2-3)	0.018
**% IL-10+ CD8+CD28- Tregs**	0 (0-0.2)	0.3 (0-1)	0.2 (0-0.5)	0.5 (0.1-0.7)	0.1 (0-0.3)	0.013
**TGF-ß1+ CD8+CD28- Treg/µl**	0.5 (0.1-2.6)	0.4 (0.1-1.5)	0.2 (0-0.5)	1 (0.2-2)	1 (0.1-1.7)	0.047
**%TGF-ß1+ CD8+CD28- Treg**	0 (0-0.1)	0 (0-0.2)	0 (0-0.1)	0.1 (0-0.2)	0.1 (0-0.2)	0.117
**Foxp3+ CD8+CD28- Tregs/µl**	0.3 (0.1-2.6)	0.5 (0-5)	0.3 (0-1)	1.3 (0-3)	0.5 (0.1-1.2)	0.25
**% Foxp3+ CD8+CD28- Tregs**	0 (0-0.1)	0 (0-0.4)	0 (0-0.2)	0.1 (0-0.3)	0.1 (0-0.1)	0.46

All values are expressed as median and Interquartile range.

% represent the proportion of cells to total lymphocytes (CD45+ cells).

### Expression of IFN-g, IL-10, TGF-ß1, and Helios in CD8+CD28- Tregs in HC, ESKD, Early and Late Recipients Tx, and STCR Patients

Opposite to IL-10 and IFN-g expression in CD4+CD25+CD127-Foxp3+ Tregs, IL-10 and IFN-g-producing CD8+CD28- Tregs were infrequent and represented rare events in all study groups (**Table 4**). Likewise, CD8+CD28- Tregs that exhibit TGF-ß1 and Foxp3 occurred rarely in all study groups.

### CD19+IL-10+ Bregs in HC, ESKD, Early and Late Recipients Tx, and STCR Patients

**Figure 4:**
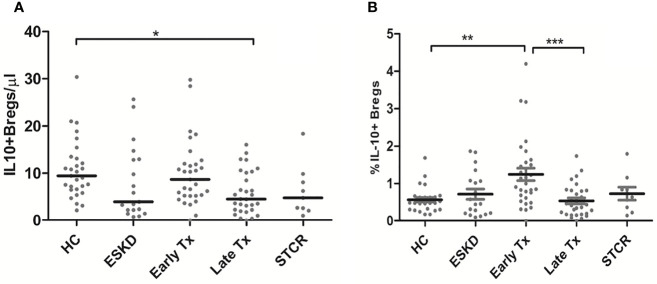
Comparison of the absolute CD19+IL-10+ Breg counts **(A)** and the percentage of CD19+IL-10+ Bregs within the CD45+ lymphocytes pool **(B)** among healthy controls (HC), end-stage kidney disease on regular dialysis (ESKD), early and late transplant recipients (Tx), and transplant recipients with a steroid-treated acute rejection (STCR): Medians were compared among the groups with Kruskal-Wallis test and p-values were adjusted after correction for multiple comparisons, *p < 0.05, **p < 0.01, ***p < 0.001.

Although early Tx recipients showed higher CD19+IL-10+ Bregs than late transplant recipients, this did not remain significant after correction for multiple comparisons (unadjusted *p* = 0.01, adjusted *p* = 0.09). The absolute IL-10+ Breg count was significantly lower in the late Tx recipients than in the HC (*p* =0.011) (**Figure 4A**). Contrary to the relative CD4+CD25+CD127-Foxp3+ Treg and CD8+CD28- Treg counts, which did not differ significantly between early and late Tx recipients, CD19+IL-10+ Breg cell relative counts were significantly higher in early than in late Tx recipients (*p*= 0.001) (**Figure 4B**).

### Perforin and Granzyme B Expression in CD8+ T Cells in HC, ESKD, Early and Late Recipients Tx, and STCR Patients

Perforin+ CD8+ T cells ranged from 0.5 – 2.5% of total CD45+ lymphocytes, whereas granzyme B+ CD8+ T cells ranged from 3.5 to 16% of total CD45+ lymphocytes in the study groups. Early Tx recipients showed significantly lower absolute perforin+ CD8+ T cells than HC and late Tx recipients (*p*=0.04; *p*=0.001, respectively). Likewise, early Tx recipients showed significantly lower absolute granzyme B+ CD8+ T cell counts than late Tx recipients (*p*<0.001). These findings might suggest a negative impact of the high doses of immunosuppressants used during the early phase of rena transplantation on perforin+ and granzyme B+ CD8+ T cells.

### Comparison of CD4+CD25+CD127-Foxp3+ Tregs, CD8+CD28- Tregs, IL-10+ Bregs, Perforin+ and Granzyme B+ CD8+ T Cells in ESKD Patients and Early Tx Recipients According to the Type of Antibody Induction (Basiliximab *vs.* rATG)

**Figure 5:**
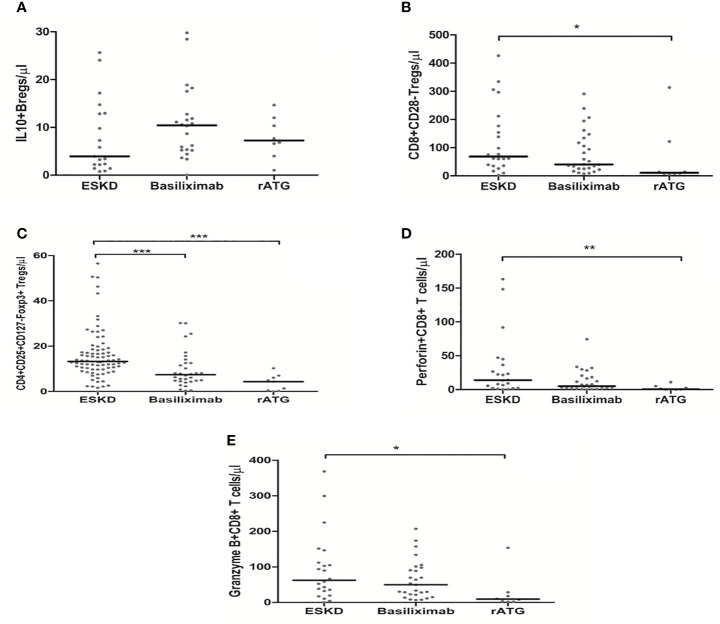
Comparison of the absolute counts of CD19+IL-10+ Bregs **(A)**, CD8+CD28- Tregs **(B)**, CD4+CD25+CD127-Foxp3+ Tregs **(C)**, perforin+ CD8+ T cells **(D)**, and granzyme B+ CD8+ T cells **(E)** among the ESKD patients and early Tx recipients, who received antibody induction with either basiliximab or rabbit ATG (rATG): Medians were compared among the groups with Kruskal-Wallis test and p-values were adjusted after correction for multiple comparisons, *p < 0.05, **p < 0.01, ***p < 0.001.

**Figure 6:**
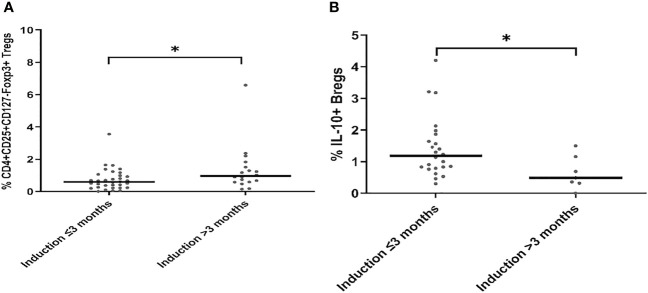
Comparison of the absolute and relative counts of CD4+CD25+CD127-Foxp3+ Tregs **(A)** and CD19+IL-10+ Bregs **(B)** in early Tx recipients, in whom antibody induction dates back either ≤ 3 months or > 3 months prior to blood sampling. Medians were compared with Mann-Whitney test, *p < 0.05.

To show the potential impact of antibody induction (rATG and basiliximab) in early Tx recipients on the absolute counts of CD4+ Tregs, CD8+ Tregs, IL-10+ Bregs, and perforin+ or granzyme B+ CD8+ T cells, we compared the absolute counts of these cells with those in ESKD patients on regular dialysis, who were considered as the baseline. We found no significant difference between the ESKD, basiliximab, and rATG groups regarding the absolute numbers of IL-10+ B cells (*p* =0.269) (**Figure 5A**). CD8+CD28- Tregs were significantly lower in the rATG group than those in the ESKD group (*p* = 0.03) (**Figure 5B**), whereas no significant difference could be detected between the basiliximab and either the rATG or the ESKD group (*p* = 0.2; *p*=0.75, respectively). CD4+CD25+CD127-Foxp3+ Tregs were significantly lower in both the basiliximab and rATG groups than those in the ESKD group (*p* = 0.001; *p*<0.001, respectively), whereas no significant difference could be detected between the basiliximab and rATG groups (p = NS) (**Figure 5C**). Perforin+ CD8+ T cells were significantly lower in the rATG group than those in the ESKD group (*p* = 0.004) (**Figure 5D**), whereas we found a trend to lower perforin+ CD8+ T cell counts in the rATG than those in the basiliximab group (*p* = 0.056). Likewise, granzyme B+ CD8+ T cells were significantly lower in the rATG than in the ESKD group (*p* = 0.016) (**Figure 5E**). No significant difference regarding the absolute counts of perforin+ or granzyme B+ CD8+ T cells could be detected between the basiliximab and the ESKD groups (*p* = NS). To show whether the effect of antibody induction on CD4+CD25+CD127-Foxp3+ Tregs, CD8+CD28- Tregs, and IL-10+ Bregs differed in relation to time post-transplantation in the early Tx group, we separated the early Tx recipients into 2 groups according to whether the antibody induction dated back maximally 3 months or more than 3 months prior to blood sampling (**Table 5**). We found that the relative CD4+CD25+CD127-Foxp3+ Treg counts were significantly lower in the group which received the antibody induction equal to or shorter than 3 months prior to blood sampling than the group that received the antibody induction more than three months prior to blood sampling (*p* = 0.034) (**Figure 6A**). Contrary to this, the relative IL-10+ Breg counts were significantly higher in the group that received the antibody induction equal to or shorter than 3 months prior to blood sampling than the group that received the antibody induction longer than 3 months prior to blood sampling (*p* = 0.022) (**Figure 6B**). We found no difference between the two groups regarding the absolute or relative CD8+CD28- Tregs (*p* = 0.66; *p* = 1, respectively). This finding suggests that CD4+ Tregs tend to increase a few months post-transplantation as the effect of antibody induction elapses, while IL-10+ Bregs tend to decrease few months post-transplantation, which suggests that IL-10+ Bregs are less, if at all, affected by antibody induction (basiliximab or rATG). Taken together, these findings suggest that CD4+CD25+CD127-Foxp3+ Tregs are negatively influenced by either rATG or basiliximab. These negative effects tend to wane few months post transplantation, whereas IL-10+ Bregs remain stable directly after Tx and tend to decrease a few months post-transplantation irrespective of antibody induction. CD8+CD28- Tregs, perforin+, and granzyme+ CD8+ T cells decrease secondary to ATG but not basiliximab.

**Table 5:** Comparison of the absolute and relative counts of CD4+CD25+CD127-Foxp3+ Tregs, CD8+CD28- Tregs, and IL-10+ Bregs in early Tx recipients in whom antibody induction dates back ≤ 3 months *vs.* > 3 months prior to blood sampling.

	Antibody induction before <3 months	Antibody induction before >3 m	P-value
**CD4+ Tregs/µl**	6 (3 – 10) (n=33)	7 (4 – 22) (n=18)	0.168
**% CD4+ Tregs**	0.6 (0.2 – 1) (n=33)	0.9 (0.6 – 1.6) (n=18)	**0.03**
**CD8+CD28- Tregs**	40 (13 – 137) (n=26)	26 (15 – 90) (n=10)	0.66
**% CD8+CD28- Tregs**	4.6 (2 – 10) (n=26)	5 (2 – 15) (n=10)	1
**CD19+IL-10+ Bregs/µl**	10 (6 – 12) (n=24)	5 (3 – 6) (n=7)	0.06
**% CD19+IL-10+ Bregs**	1.2 (0.8 – 1.8) (n=24)	0.5 (0.3 – 1) (n=7)	**0.022**

All values are expressed as median and Interquartile range.

% represent the proportion of cells to total lymphocytes (CD45+ cells).

Bold values denote statistically significant comparisons.

### Association Between Standard Immunosuppressive Drugs and CD4+CD25+CD127-Foxp3+ Tregs in Early Tx, Late Tx, and STCR Patients

**Figure 7:**
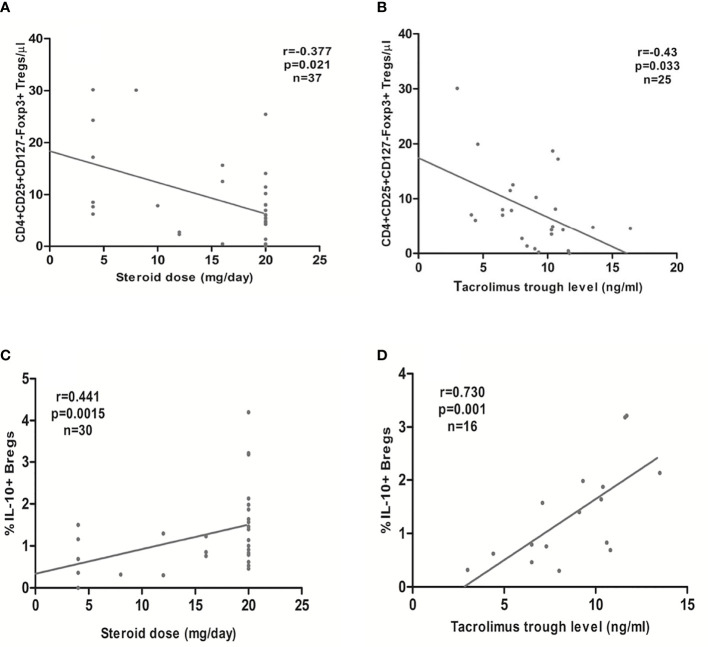
Association between the steroid daily dose or tacrolimus trough levels in early transplant recipients and the absolute CD4+CD25+CD127-Foxp3+ Tregs (**A**, **B**, respectively) or the relative CD19+IL-10+ Breg count (percentage of CD19+IL-10+ to the total CD45+ lymphocyte pool) (**C**, **D**, respectively): CD4+CD25+CD127-Foxp3+ Tregs showed a significant inverse association with steroid daily dose and tacrolimus trough levels, whereas CD19+IL-10+ Bregs showed a significant positive association with steroid daily dose and tacrolimus trough levels.

The steroid daily dose and tacrolimus trough levels showed a significant negative association with the absolute CD4+CD25+CD127-Foxp3+ Treg counts in the early Tx recipients (r= -0.377, *p* =0.021; r = -0.428, *p* = 0.033, respectively) (**Figures 7A, B**). No significant correlation between the mycophenolate dose and the absolute CD4+CD25+CD127-Foxp3+ Treg counts could be detected (r= 0.13, *p* =0.44). In multiple regression analysis, the absolute Treg counts remained significantly inversely associated with the steroid dose in early Tx recipients (standardized coefficient beta = -0.543, CI (-1.1 - -0.32), *p* = 0.001) (**Table 6**).

**Table 6:** Multiple linear regression analysis of the factors affecting absolute CD4+CD25+CD127-Foxp3+ Treg counts in early renal transplant recipients.

Variables	Unstandardized B (95% CI) (n=37)	Standardized beta	*p*-value	
**Age**	-0.152 (-0.39- 0.086)	-0.207	0.20	excluded
**Sex (males vs. females)**	-0.375 (-5.9 – 5.1)	-0.023	0.89	excluded
**Days post-transplantation**	-0.021 (-0.06- 0.02)	-0.181	0.323	excluded
**Living vs deceased donor**	-1.1 (-16 - 13)	-0.039	0.876	excluded
**Cold ischemia time (hours)**	-0.003 (-0.01- 0.009)	-0.1	0.64	excluded
**Induction (Basiliximab vs. rATG)**	-3.8 (-9.7 – 2.1)	-0.20	0.19	excluded
**Cyclosporine vs. tacrolimus**	2.6 (-3.6 – 9)	0.17	0.37	excluded
**Daily mycophenolate dose (mg)**	-0.003 (-0.014 – 0.009)	-0.074	0.63	excluded
**Daily steroid dose (mg)**	-0.74 (-1.1 - -0.32)	-0.54	**0.001**	

Bold values denote statistically significant comparisons.

In the late Tx patients, we found no significant correlation between the steroid dose, mycophenolate dose, tacrolimus or cyclosporine trough levels and the absolute CD4+CD25+CD127-Foxp3+ Treg counts (r =0.23, *p=*0.10; r=-0.005, *p*=0.98; r=-0.023, *p* = 0.86; r=-0.25, *p* = 0.33, respectively).

In the STCR patients, no significant correlation between the absolute CD4+CD25+CD127-Foxp3+ Treg cell count and steroid or mycophenolate dose could be detected (r= -0.453, *p* = 0.233, n=9; r = 0.252, *p* =0.547, n=8, respectively). Likewise, we found no significant association between the absolute or relative CD4+CD25+CD127-Foxp3+ Treg counts and cyclosporine (r= 0.2, *p* =0.8, n=4; r= 0.8, *p* = 0.2, n=4, respectively) or tacrolimus trough levels (r= -0.8, *p* = 0.2, n= 4; r = -0.6, *p* = 0.4, n=4, respectively).

### Association Between Standard Immunosuppressive Drugs and CD8+CD28- Tregs in Early Tx, late Tx, and STCR Patients

In multiple regression analysis, we found no significant association between the type of antibody induction (basiliximab *vs*. rATG) and the absolute count of CD8+CD28- Tregs after adjustment for possible confounding factors (unstandardized coefficient beta = 47.5, CI (-19 – 114), *p* = 0.155) (**Table 7**). We found no significant association between either the absolute or relative CD8+CD28- Tregs and the steroid or mycophenolate dose or tacrolimus or cyclosporine trough levels either in early or late Tx recipients (*p* = NS). Likewise, we found no correlation between the absolute or relative CD8+CD28- Tregs and the type of antibody induction (rATG *vs.* basiliximab), steroid or mycophenolate dose, and tacrolimus or cyclosporine trough levels in STRC patients (*p* = NS). CD8+CD28- Tregs were, however, independently negatively associated with age in early Tx recipients (unstandardized coefficient beta = 2.5, CI (0.24 – 4.9), *p* = 0.03).

**Table 7:** Multiple linear regression analysis of the factors affecting absolute CD8+CD28- Treg counts in early renal transplant recipients.

Variables	Unstandardized B (95% CI) (n=36)	Standardized beta	*p*-value	
**Age**	2.5 (0.24 - 4.9)	0.381	**0.03**	
**Sex (males *vs.* females)**	5.7 (-52 – 64)	0.037	0.84	excluded
**Days post-transplantation**	-0.003 (-0.5- 0.5)	-0.003	0.99	excluded
**Living *vs* deceased donor**	-22.8 (-149 - 104)	-0.088	0.714	excluded
**Cold ischemia time (hours)**	-0.07 (-0.15 - 0.015)	-0.28	0.104	excluded
**Induction (Basiliximab vs. ATG)**	47.5 (-19 – 114)	0.27	0.15	excluded
**Cyclosporine *vs.* tacrolimus**	21.7 (-42 – 86)	0.144	0.49	excluded
**Daily mycophenolate dose (mg)**	0.01 (-0.14 – 0.16)	0.032	0.89	excluded
**Daily steroid dose (mg)**	-0.67 (-5.7- 4.3)	-0.05	0.78	excluded

Bold values denote statistically significant comparisons.

### Association Between Standard Immunosuppressive Drugs and CD19+IL-10+ Bregs in Early Tx, Late Tx, and STCR Patients

In the early Tx recipients, we found a significant positive correlation between the relative CD19+IL-10+ Breg counts and both the steroid dose and tacrolimus trough levels (r= 0.441, *p* = 0.015, n=30; r= 0.73, *p* = 0.001, n=16, respectively) (**Figures 7C, D**). In multiple regression analysis, the relative CD19+IL-10+ Breg counts remained significantly positively associated with the steroid dose after adjustment for possible confounding factors (standardized coefficient beta = 0.43, CI (0.01- 0.127), *p* = 0.024) (**Table 8**).

**Table 8:** Multiple linear regression analysis of the factors affecting relative CD19+IL-10+ Breg counts in early renal transplant recipients.

Variables	Unstandardized B (95% CI) (n=30)	Standardized beta	*p*-value	
**Age**	0.013 (-0.02 – 0.046)	0.15	0.43	
**Sex (males *vs.* females)**	-0.105 (-1 – 0.8)	-0.055	0.8	excluded
**Days post-transplantation**	0.01 (-0.014 - 0.033)	0.45	0.411	excluded
**Living *vs* deceased donor**	0.56 (-1 – 2.1)	0.19	0.47	excluded
**Cold ischemia time (hours)**	0.001 (0 - 0.002)	0.41	**0.033**	
**Induction (Basiliximab *vs.* rATG)**	0.128 (-1 – 1.3)	0.062	0.82	excluded
**Cyclosporine *vs.* tacrolimus**	-0.171 (-1.2 – 0.8)	-0.09	0.72	excluded
**Daily mycophenolate dose (mg)**	0 (-0.002 – 0.001)	-0.11	0.64	excluded
**Daily steroid dose (mg)**	0.068 (0.01 – 0.127)	0.435	**0.024**	

Bold values denote statistically significant comparisons.

In late Tx recipients, we found no significant correlation between the absolute or relative CD19+IL-10+ Bregs and steroid dose (r= -0.135, *p* =0.48; r= -0.135, *p* = 0.478, respectively). We found, however, a significant inverse association between cyclosporine trough levels and absolute and relative CD19+IL-10+ Breg counts (r= -0.66, *p* = 0.014, n =13; r = -0.82, *p* = 0.001, n=13, respectively). We found no significant association between the absolute or relative CD19+IL-10+ Bregs and either mycophenolate dose or tacrolimus trough levels (*p*= NS).

In STCR patients, we found no significant correlation between the absolute or relative CD19+IL-10+ Bregs and the type of antibody induction, steroid or mycophenolate dose, and tacrolimus or cyclosporine trough levels (*p* = NS).

### Association Between Standard Immunosuppressive Drugs and Perforin- or Granzyme B-Expressing CD8+ T Cells in HC, ESKD, Early Tx, Late Tx, and STCR Patients

We found no association between the absolute or relative perforin+ or granzyme B+ CD8+ T cells and the steroid dose, mycophenolate dose, and cyclosporine or tacrolimus trough levels either in early Tx, late Tx, or STCR patients (*p* = NS).

### Association Between CD4+CD25+CD127-Foxp3+ Tregs, CD8+CD28- Tregs, and CD19+IL-10+ Bregs in Early Tx, Late Tx, and STCR Patients 

We found no significant association between the absolute or relative CD4+CD25+CD127-Foxp3+ Treg counts and the absolute or relative CD8+CD28- Treg counts in early or late Tx patients, respectively (r=0.052, *p*=0.76; r= -0.056, *p* = 0.74; r=0.155, *p*=0.311; r= -0.23, *p*=0.126, respectively). Likewise, we found no significant association between the absolute or relative CD4+CD25+CD127-Foxp3+ Treg counts and the absolute or relative CD8+CD28- Treg counts in STCR patients, respectively (r= 0.133, *p*=0.73, n=9; r=-0.56, *p*=0.112, n=9, respectively).

We found a significant correlation between the absolute Treg counts and relative CD19+IL-10+ Breg counts in early Tx recipients (r=-0.433, *p*=0.015, n=31) (**Figure S1**). This might be attributed to the differential effect of immunosuppressive drugs, particularly steroids, on CD4+CD25+CD127-Foxp3+ Tregs and CD19+IL-10+ Bregs. As previously mentioned, steroid dose was positively associated with the relative count of CD19+IL-10+ Bregs (**Figure 7C**) and negatively associated with the absolute CD4+CD25+CD127-Foxp3+ Treg counts in early Tx recipients (**Figure 7A**). Contrary to early Tx patients, in late Tx patients the absolute CD19+IL-10+ Bregs were positively associated with the absolute CD4+CD25+CD127-Foxp3+ Treg counts (r=0.601, *p*<0.001). This might be attributed to the relative decrease of Bregs and the constantly low Tregs in late Tx compared with early Tx recipients under the effect of decreased dose of immunosuppressive drugs, particularly steroids.

We also studied the association between CD8+CD28- Tregs and CD19+IL-10+ Bregs in Tx recipients. We found a significant negative association between the relative CD8+CD28- Treg and CD19+IL-10+ Breg counts only in late Tx recipients (r=-0.364, *p*=0.048, n=30). The underlying cause of this negative association might be attributed to the relative increase in CD8+CD28- Tregs and the relative decrease of CD19+IL-10+ Bregs in late Tx recipients (**Table 2**). Likewise, In STCR patients, the relative CD19+IL-10+ Breg counts were negatively associated with the relative CD8+CD28- Treg counts (r= -0.717, *p*= 0.03, n=9).

### Association of CD4+CD25+CD127-Foxp3+ Tregs, CD8+CD28- Tregs, and CD19+IL-10+ Bregs With the Glomerular Filtration Rate and C-Reactive Protein (CRP) in Early Tx, Late Tx, and STCR Patients

We found no significant association between the absolute or relative CD4+CD25+CD127-Foxp3+ Treg, CD8+CD28- Treg, or CD19+IL-10+ Breg counts and the GFR (ml/min) or CRP (mg/l) in early or late Tx recipients (*p*=NS). We found a significant positive association between the absolute CD4+CD25+CD127-Foxp3+ Treg counts and GFR but not CRP in STCR patients (r=0.683, *p*=0.04). This can be probably ascribed to the decrease in Tregs secondary to pulse steroids and the lag in the increase or the incomplete response of GFR shortly after rejection treatment. We found no significant correlation between the absolute or relative CD8+CD28- Tregs or CD19+IL-10+ Bregs and GFR or CRP in STCR patients (*p*=NS).

## Discussion

CD4+CD25+CD127-Foxp3+ Tregs are known for their powerful immunosuppressive potential. Gaps still exist regarding further characterization of CD4+ Tregs as well as regarding their interaction with other regulatory cells, including CD8+ Tregs and Bregs. In the current study, we aimed to analyze the expression of inhibitory cytokines, including TGF-ß1 and IL-10, in CD4+ and CD8+ Tregs that partially mediate the suppressive function of Tregs. We also analyzed the frequencies of CD4+ and CD8+ Tregs in ESKD patients, early and late stable Tx recipients, and STCR patients. In addition, we aimed to analyze the changes in CD8+CD28- Tregs and IL-10+ Bregs that occur parallel to the changes in CD4+ Tregs in the study groups, particularly in relation to standard antibody induction and maintenance immunosuppressive drugs. Furthermore, we analyzed the association between CD4+CD25+CD127-Foxp3+ Tregs, CD8+CD28- Tregs, and IL-10+ Bregs in the aforementioned study groups. Finally, we analyzed the association between CD4+ Tregs, CD8+ Tregs, or IL-10+ Bregs and clinical parameters, including GFR and CRP in Tx recipients.

Production of inhibitory cytokines, such as IL-10, TGF-ß1, and IL-35, contributes to the mechanisms that mediate the suppressive effect of CD4+CD25+CD127+Foxp3+ Tregs (11, 12). Foxp3- CD4+ Tregs expressing IL-10 have been termed Tr1 cells, whereas TGF-ß1 producing CD4+ Tregs have been termed Th3 cells (13, 14). Both Foxp3- and Foxp3+ thymic “tTregs” and peripheral Tregs “pTregs” can produce IL-10 (15, 16). IL-10-knockout mice did not develop spontaneous autoimmunity but rather immune dysregulation at the body’s environmental interfaces in the form of colitis and lung inflammation (15). Our study showed that the majority of CD4+CD25+CD127-Foxp3+ Tregs express IL-10, regardless of whether these cells were analyzed in HC, ESKD patients, or renal Tx recipients. This finding suggests that IL-10 production might be one of the important mechanisms of action of Tregs in humans. TGF-ß is a cytokine with pleiotropic effects, depending on the biological milieu and interacting cells. TGF-ß production and secretion by Tregs play a pivotal role in *in vivo* immunosuppression. Tregs exert contact-dependent immunosuppression through TGF-ß1 production. TGF-ß is present in an active form on the surface of Tregs bound to a membrane protein called GARP, which contributes to the activation of TGF-ß1, and inhibition of GARP through blocking antibodies was shown to result in inhibition of human Tregs (17). Supporting the importance of TGF-ß1 production for the function of Tregs, Turner et al. reported that “haploinsufficiency” of TGF-ß1 in Tregs led to a reduction in RORγt+ Tregs, which subsequently resulted in increased sensitivity to food allergy (18). Moreover, another study showed that TGF-ß expression in CD4+CD25+ Tregs was crucial to sustaining Foxp3 expression and maintaining their regulatory function (19). In accordance with this, a study showed the pivotal role of renal allograft infiltrating Foxp3+ Tregs expressing IL-10, TGF-ß1, and IFN-g in a spontaneous allograft tolerance model in mice (20). Studies investigating the expression of IFN-g and anti-inflammatory cytokines, including IL-10 and TGF-ß, in Tregs are scarce. Our study showed that the majority of CD4+Foxp3+ and CD8+CD28- Tregs did not produce TGF-ß1.

Helios, a transcription factor of the Ikaros family, has long been suggested as a marker to differentiate between tTregs and pTregs (3). This suggestion has been contradicted by both Vehagen and Wraith and Gottschalk et al., who showed that *in vitro*-induced Tregs (iTregs) can express Helios upon stimulation with antigen presenting cells (APC) (2, 21). Furthermore, pTregs have been shown to express Helios upon low dose antigen stimulation in the absence of adjuvant (2). Recently, it has been reported that Helios+ CD4+Foxp3+ Tregs are associated with more stability and suppressive potential than Helios- Foxp3+ Tregs (4). Moreover, Helios+ Tregs showed more stable Foxp3 expression compared with Helios- Tregs when transferred to lymphopenic mice. Gottschalk et al. were unable to detect Helios expression in CD4+Foxp3- T cells, even after their activation (4). Taken together, these findings suggest Helios as a marker of Treg stability and might indicate a thymic origin. In the current study, around one-half of CD4+CD25+CD127-Foxp3+ Tregs in the peripheral blood of HC, ESKD, and transplant recipients expressed Helios, suggesting their stability. Helios+ Tregs were lower in late Tx recipients compared with ESKD patients. This might be attributed to the state of immune quiescence of the late Tx recipients in our study. Since Tregs represent a subset of cells that are activated to suppress overreacting effector cells in the setting of inflammation, they are not expected to increase under stable conditions. In a previous study, we found that Helios+ and Helios- Tregs lacking IFN-g production predominate in the peripheral blood of long-term transplant recipients (22). IFN-g producing CD4+Foxp3+ Tregs cells were lower in both early and late Tx patients compared with HC. IFN-g+ CD4+Foxp3+ Tregs are believed to be the first line of peripherally induced or thymic activated Tregs that can specifically or unspecifically inhibit effector cells *via* multiple mechanisms, including the production of IL-10 and TGF-ß (23). Based on the above observations, in other studies and in ours, we can conclude that CD4+Foxp3+ Tregs in HC, ESKD, and Tx recipients exert their function partially through production of IL-10, TGF-ß, and IFN-g. In addition, we assume that at least one-half of the CD4+CD25+CD127-Foxp3+ Tregs represents stable Tregs with a powerful suppressive potential.

In the current study, we found that the antibody induction affected CD4+CD25+CD127-Foxp3+ Tregs, CD8+CD28- Tregs, and IL-10+ Bregs differently during the early Tx phase. CD4+CD24+CD127-Foxp3+ Tregs seem to have been suppressed by both rATG and basiliximab. Contrary to this, CD8+CD28- Tregs were significantly lower only in the rATG than in the ESKD group. IL-10+ Bregs were comparable among the ESKD, rATG, and basiliximab groups. Valdez-Ortiz et al. showed that rATG induced a significant proliferation of CD4+CD25+Foxp3+ Tregs in peripheral blood of transplanted mice compared to mice not treated with rATG (24). After extraction of these *in vivo*-induced Tregs, they were applied to a mixed lymphocyte co-culture, pre-stimulated with anti CD3 and anti CD28 antibodies, to observe their suppressive capacity. Interestingly, both rATG and non-rATG-induced Tregs could suppress CD8+ T cell proliferation *in vitro*. The suppressive effect, however, was higher with rATG-induced Tregs compared with non-rATG-induced Tregs. A major shortcoming of the latter study is the absence of a direct comparison between rATG, which is used in patients with high immunologic risk in the *in vivo* settings, and the commonly used antibody induction immunosuppressive drug basiliximab, regarding their effect on the suppressive capacity of CD4+CD25+Foxp3+ Tregs.

The effect of steroids on CD4+CD25+CD127-Foxp3+ Tregs is controversially discussed in the literature. Seissler et al. reported that the total peripheral blood CD4+CD127low/- Foxp3+ Tregs within the CD4+ T cell pool were not affected by steroid pulse therapy. The authors showed, however, a shift in composition of CD4+ T cells to favor the highly suppressive DR^high^ CD45RA^-^ Tregs. This stimulatory effect of steroid pulse therapy on DR^high^ CD45RA^-^ Tregs was temporary and limited to the duration of administration (25). The gating strategy in this study, however, differed from the consensus definition of Tregs, which are distinguished from other cells by the marker combination CD4+CD25highCD127-Foxp3+. Another study showed that short-term administration of high-dose intravenous prednisolone followed by gradual tapering to low dose oral steroid in immunocompetent patients did not cause an increase of the percentage of CD4+ Tregs within the CD4+ T cell pool. Again, many marker combinations were used to define the CD4+ Treg pool in that study (26). In the current study, we found that in early Tx recipients, the absolute CD4+Foxp3+ Treg counts were independently negatively associated with steroid dose. We also evaluated the absolute and relative frequencies of CD4+CD25+CD127-Foxp3+ Tregs in renal transplant recipients with a biopsy-proven cellular rejection, who received steroid pulse therapy 1 week - 3 months before blood sampling. We found that the absolute CD4+Foxp3+ Treg counts were significantly lower in these patients compared with HC and ESKD patients, supporting a negative impact of steroids on conventional CD4+ Tregs.

Like CD4+CD25+CD127-Foxp3+ Tregs, Bregs can mitigate autoimmune diseases, suppress overreacting effector cells, and prevent allograft rejection. Although no unifying marker combination has been used to date for defining Bregs, IL-10-producing B cells have been conventionally regarded as Bregs. In the current study, we found that IL-10+ Bregs behave contrary to CD4+Foxp3+ Tregs in renal Tx recipients. Whereas CD4+Foxp3+ Tregs decrease early after transplantation and tend to increase thereafter, IL-10+ Bregs in early Tx recipients, though statistically comparable to those in the ESKD patients, are higher than in late Tx recipients. This opposing behavior might be the cause of the significant inverse association of IL-10+ Bregs with CD4+CD25+CD127-Foxp3+ Tregs shown in our study. Standard antibody induction and maintenance immunosuppression applied in the setting of renal Tx play a key role in the differences regarding the frequencies of CD4+CD25+CD127-Foxp3+ Tregs, CD8+CD28- Tregs, and IL-10+ Bregs. Our findings support a differential effect of antibody induction immunosuppressants, steroid dose, and tacrolimus trough levels on CD4+Foxp3+ Tregs and IL-10+ Bregs. CD4+CD25+CD127-Foxp3+ Tregs were lower in both the basiliximab and rATG groups in early Tx recipients compared with those in ESKD patients, whereas IL-10+ Bregs showed comparable absolute counts among the ESKD, basiliximab, and rATG groups. In addition, our results showed that CD4+CD25+CD127-Foxp3+ Tregs tend to increase a few months post transplantation in relation to the waning effect of antibody induction, opposite to IL-10+ Bregs, which were significantly lower 3 months post Tx compared to their relative counts shortly after Tx. Moreover, in multiple regression analysis, we found that in early Tx recipients, the absolute CD4+Foxp3+ Treg counts were independently negatively associated with the steroid dose. Opposite to this, multiple regression analysis showed that IL-10+ Bregs were independently positively associated with the steroid dose in early Tx recipients. Furthermore, and opposite to CD4+Foxp3+ Tregs, IL-10+ Bregs showed a positive correlation with tacrolimus trough levels in early Tx recipients. This finding might indicate that IL-10+ Bregs represent a subset of regulatory cells that are less affected by standard immunosuppression used early post renal transplantation. In late renal Tx recipients, we found a significant inverse correlation between the cyclosporine trough levels and IL-10+ Bregs. A negative impact of the lower doses of cyclosporine used in late transplantation seems less likely in the light of the positive association between higher CNI trough levels (tacrolimus) and IL-10+ Bregs in early Tx recipients. A possible explanation for this inverse relationship in late Tx recipients is the disproportionate decrease of cyclosporine trough levels compared with that of IL-10+ Breg count in late Tx recipients. Although Bregs have been conventionally defined through their expression of IL-10, this assumption has been recently contradicted by Lighaam et al., who reported that *in vitro*-induced IL-10+ B cells co-expressed the proinflammatory cytokines IL-6 and/or TNF-alpha (27). Furthermore, a large proportion of these IL-10+ B cells lost their IL-10 expression after culture and restimulation, suggesting their plasticity. Whether this assumed *in vitro* plasticity of IL-10+ Bregs applies to *in vivo* settings, or whether their expression of proinflammatory cytokines entails an inflammatory potential is still unknown.

CD8+CD28- T cells originate in the thymus (28). They were shown to possess an immunosuppressive potential. In experimental autoimmune encephalomyelitis, CD8+CD28- Tregs inhibited the secretion of IFN-g by myelin oligodendrocyte glycoprotein-specific CD4+ T cells (29). It was shown that absent expression of CD28 in human lymphocytes is related to either cell senescence or sustained exposure to antigens (28). Hence, CD8+CD28- Tregs were found to occur in higher frequencies in the elderly as well as under chronic inflammatory conditions. In line with this, we found that CD8+CD28- Tregs were independently positively associated with the age in early transplant recipients. Assadiasl et al. reported significantly higher CD8+CD28- Tregs in long-term stable Tx-recipients compared with those in HC and renal transplant recipients with a biopsy-proven chronic allograft nephropathy (30). They concluded that CD8+CD28- Tregs might promote renal allograft survival. Another study showed that renal transplant recipients with acute rejection within 6 months post-transplantation had lower CD8+CD28-Foxp3+ Tregs on the 14^th^ day post transplantation compared with those with no rejection (31). In the present study, we found comparable percentages of CD8+CD28- Tregs within the CD8+ T cell pool in HC, ESKD patients, early and late stable Tx recipients, and STCR patients. To the best of our knowledge, the effect of standard immunosuppressants on CD8+CD28- Tregs has been scarcely, if at all, studied in renal transplant recipients. We found that the absolute CD8+CD28- Treg counts in the rATG group in early Tx recipients were lower than those in the ESKD group. CD8+CD28- Treg counts did not differ significantly between the ESKD and basiliximab groups. An *in vitro* study showed that CD8+CD28- Tregs are resistant to methylprednisolone (32). In line with that, we found no significant association between the absolute or relative CD8+CD28- Tregs and the dose of steroids in Tx recipients. Moreover, we found no significant association between these cells and either mycophenolate dose or trough levels of calcineurin inhibitors (tacrolimus and cyclosporine) in both early and late Tx recipients. Unlike CD4+Foxp3+ Tregs, CD8+CD28- Tregs expressed TGF-ß1, IL-10, INF-g, and Foxp3 infrequently. In accordance with this, Fenoglio et al. demonstrated that CD8+CD28- Tregs lack Foxp3 expression *in vitro*. Interestingly, the same study showed that *in vitro*-generated CD8+CD28- Tregs were anergic to a variety of mitogens and stimulating cytokines, including IL-2 (32). These CD8+CD28-Foxp3- Tregs are non-antigen-specific and lack the expression of CD127 and CD56 (32). Another study investigated the expression of Foxp3 in CD8+CD28- Tregs in the peripheral blood of healthy volunteers as well as in allospecific T cell lines (TCL) by use of RT-PCR. CD8+CD28- Tregs extracted from the peripheral blood of healthy volunteers lacked the expression of Foxp3, whereas the antigen-specific CD8+CD28- Tregs from TCL expressed Foxp3 (33). It was shown that *in vitro* stimulation of CD8+CD28- Tregs led to production of IL-10 and TGF-ß (34). Filaci et al. showed that IL-10-producing CD8+CD28- Tregs can inhibit cytotoxic T cells through down-regulation of MHC I expression on the target cells (35). Another study reported that CD8+CD28- Tregs exert their immunosuppressive function through direct contact with effector cells or with antigen presenting cells (36). Furthermore, CD8+CD28- Tregs were found to exert their function by rendering APCs tolerogenic with a subsequent inhibition of CD4+ T cells (7). Taken together and based on the infrequent occurrence of IL-10, IFN-g, and TGF-ß-producing CD8+CD28- Tregs, it might be intuitive that CD8+CD28- Tregs act primarily through direct cell-cell contact or through induction of tolerance in APCs in ESKD patients and in renal transplant recipients. In the present study, we found that the relative counts of CD8+CD28- Tregs were inversely associated with those of IL-10+ Bregs in late Tx recipients. This might be attributed to the observation that IL-10+ Bregs tend to decrease with time post-transplantation, whereas CD8+CD28- Tregs tend to increase late post-transplantation, possibly because of the reduced doses of immunosuppressants and the elapsed effect of the antibody induction.

CD8+ T cells could be detected in allografts during acute rejection (37). Cytotoxic CD8+ T cells exert their function mainly through secretion of perforin and granzyme, which induce pore formation in and apoptosis of the allograft cells, respectively. In the current study, we found that early transplant recipients showed lower absolute counts of perforin+ and granzyme+ CD8+ T cells than late Tx recipients. Moreover, basiliximab was associated with higher absolute counts of these cells than rATG. Taken together, these findings suggest that perforin+ and granzyme B+ CD8+ T cells decrease in the early transplant period, possibly due to the effect of antibody induction. Our results did not show a significant correlation between these cells and either CD4+CD25+CD127-Foxp3+ Tregs, CD8+CD28- Tregs, and IL-10+ Bregs, possibly due to the clinical stability of the study patients at the time of blood sampling. Since regulatory cells come to action mainly to suppress overreacting effector cells, it might be intuitive that an association between perforin+ and/or granzyme B+ CD8+ T cells and Bregs, CD4+ Tregs, or CD8+ Tregs might be revealed during allograft rejection before application of anti-rejection medications.

Lastly, we found no significant association between the CD4+CD25+CD127-Foxp3+ Treg, CD8+CD28- Tregs, and IL-10+ Bregs and GFR or CRP in early or late Tx recipients. This finding suggests that the counts of the regulatory cells estimated in our study are unlikely to have been affected by inflammatory conditions. Moreover, it might imply that estimation of Treg and Breg counts in a state of immune quiescence is unlikely to be able to predict graft function. In STCR patients, however, GFR was positively associated with CD4+CD25+CD127-Foxp3+ Tregs. This might be ascribed to the decline in the Treg counts and the lag in the increase or the incomplete response of GFR shortly after treatment of rejection.

In summary, our findings suggest that CD19+IL-10+ Bregs and Tregs (CD4+ and CD8+) behave in opposite ways in early transplant recipients, possibly due to the predominant negative impact of immunosuppressants on Tregs. CD19+IL-10+ Bregs seem not to be suppressed by standard immunosuppressive drugs in the early transplantation phase.

Our study has limitations. We did not include patients with acute rejection from whom blood samples were obtained before steroid pulse therapy. This might have helped reveal how the different Tregs and Bregs as well as effector perforin+ and granzyme B+ CD8+ T cells behave in relation to each other independent of the effect of high steroid doses. Although we analyzed the expression of various cytokines in CD4+CD25+CD127-Foxp3+ Tregs, which mediate the inhibitory function of these cells, we did not analyze the state of demethylation of the TSDR of CD4+Foxp3+ Tregs to reveal the frequency of peripheral tTregs. Since only very few patients in our study received inhibitors of mammalian target of rapamycin (mTOR inhibitors), we could not study the association between mTor inhibitor trough levels and the Treg or Breg counts. Lastly, significant associations between the doses or levels of immunosuppressants and regulatory cell counts in the study patients do not necessarily signify a cause-effect relationship. Conversely, absence of correlation between immunosuppressants and regulatory cells does not exclude an indirect effect of these immunosuppressants on the analyzed regulatory cells through third-party cells.

## Data Availability Statement

The datasets used during the current study are available from the corresponding author on reasonable request.

## Ethics Statement

The studies involving human participants were reviewed and approved by Heidelberg Ethical Committee (S-225/2014). The patients/participants provided their written informed consent to participate in this study.

## Author Contributions

EI, MA, and VD designed the study. EI and MA performed all experiments of the study. MA and HK recruited the study patients. MA conducted the statistical analysis. MA, EI, and VD wrote the manuscript. GO and MZ made substantial contributions to conception and design of the study. VD, GO, RW, HK, CM, MZ, GO, and NE critically revised the manuscript. RW, HK, CM, GO, and MZ have been involved in important intellectual content. All authors contributed to the article and approved the submitted version.

## Conflict of Interest

CM and GO together with the University of Heidelberg, are cofounders of TolerogenixX GmbH, a biotechnology company that holds licenses for modified immune cell (MIC) treatment. GO holds a patent for MIC treatment (“Immunosuppressive blood cells and methods of producing the same.” Patent no. WO 2010/000730, EP 2318020). CM and VD filed a patent application for MIC treatment (“MIC therapy for specific immunosuppression in transplantation” Patent no. PCT/EP2019/062857).

The remaining authors declare that the research was conducted in the absence of any commercial or financial relationships that could be construed as a potential conflict of interest.

## Supplementary Material

Supplementary Figure 1 Association between the absolute CD4+CD25+CD127-Foxp3+ Tregs and relative CD19+IL-10+ Bregs in early transplant recipients: a significant inverse correlation was found.

## Abbreviations

ESKD, end-stage kidney disease; Foxp3, forkhead box protein P3; GFR, glomerular filtration rate; IFN-g, interferon gamma; rATG, rabbit anti-thymocyte globulin; STCR, steroid-treated cellular rejection; TGF-ß, transforming growth factor beta; Tx, transplantation.
