# Supplementary figures and images for: CD4+CD25+CD127-Foxp3+ and CD8+CD28- Tregs in Renal Transplant Recipients: Phenotypic Patterns, Association With Immunosuppressive Drugs, and Interaction With Effector CD8+ T Cells and CD19+IL-10+ Bregs
Source: Front Immunol. 2021 Jul 15;12:716559. doi: 10.3389/fimmu.2021.716559 (PMC8320594; doi:10.3389/fimmu.2021.716559)

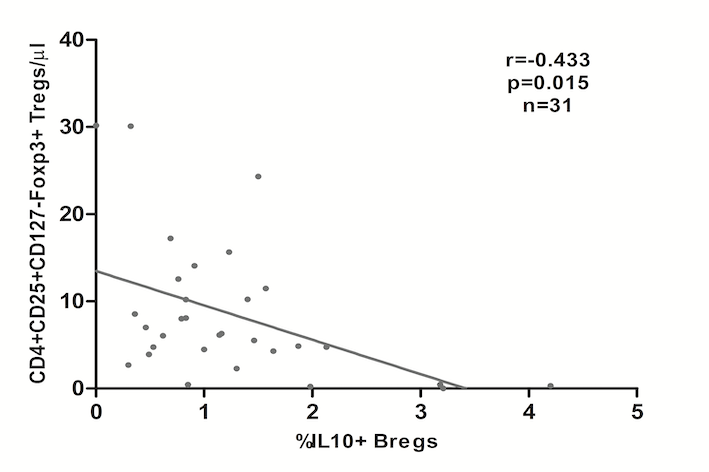

Supplement: Supplementary Figure 1 — Association between the absolute CD4+CD25+CD127-Foxp3+ Tregs and relative CD19+IL-10+ Bregs in early transplant recipients: a significant inverse correlation was found. [file Image_1.tif]
